# New therapeutic targets for pulmonary sarcomatoid carcinomas based on their genomic and phylogenetic profiles

**DOI:** 10.18632/oncotarget.24365

**Published:** 2018-01-31

**Authors:** Takahiro Nakagomi, Taichiro Goto, Yosuke Hirotsu, Daichi Shikata, Yujiro Yokoyama, Rumi Higuchi, Kenji Amemiya, Kenichiro Okimoto, Toshio Oyama, Hitoshi Mochizuki, Masao Omata

**Affiliations:** ^1^ Lung Cancer and Respiratory Disease Center, Yamanashi Central Hospital, Yamanashi, Japan; ^2^ Genome Analysis Center, Yamanashi Central Hospital, Yamanashi, Japan; ^3^ Department of Pathology, Yamanashi Central Hospital, Yamanashi, Japan; ^4^ Keio University, Tokyo, Japan; ^5^ University of Tokyo, Tokyo, Japan

**Keywords:** sarcomatoid cancer, lung cancer, mutation, next-generation sequencing, programmed death ligand-1

## Abstract

**Objectives:**

Pulmonary sarcomatoid carcinomas are rare and generally aggressive tumors composed of carcinomatous and sarcomatous components; however, the evolution of sarcomatoid cancer has not been elucidated. Here, we aimed to evaluate the mutational profiles and phylogeny of sarcomatoid carcinomas using next generation sequencing and *in-silico* analysis to facilitate the development of novel therapies.

**Methods:**

Four patients who underwent surgery for sarcomatoid cancer were enrolled. Cancer cells were collected from carcinomatous and sarcomatous components in each tumor by laser capture microdissection. Next-generation sequencing was performed in each component, and the mutation profiles were compared. For further inference of phylogenies, phylogenetic and PyClone analyses were performed. Mismatch repair disturbance and programmed death ligand-1 (PD-L1) expression were also evaluated.

**Results:**

Comparative genetic analysis of different histological areas revealed that the separate components shared several common mutations, which showed relatively high cellular prevalence in the PyClone statistical inference. Phylogenetic analysis showed that the sarcomatous component had ramified from the carcinomatous component in the early phase of the evolution process and accumulated a number of mutations that were different from those of the carcinomatous component. Moreover, microsatellite instability was detected in a case of sarcomatoid cancer and PD-L1 was strongly positive (≥ 50%) in all sarcomatoid cancers.

**Conclusions:**

Our data suggest that sarcomatoid carcinoma evolves from a common ancestral clone, and its phylogenetic features may reflect high-grade malignancy in pulmonary sarcomatoid carcinoma. High tumor mutation burden and strong PD-L1 staining may provide a rationale for the use of targeted immunotherapies in pulmonary sarcomatoid carcinomas.

## INTRODUCTION

Pulmonary sarcomatoid carcinomas are rare tumors, representing only 0.3–1.3% of non-small cell lung cancer cases [1]. These carcinomas are defined as a small subgroup of poorly differentiated tumors containing sarcoma-like elements. They are characterized by poor prognosis and resistance to conventional platinum-based chemotherapy and radiotherapy [2, 3]. Furthermore, the prognosis is often poor, irrespective of the stage of disease [2, 3]. Little is known about the biology of these neoplasms or the mechanisms of chemo- or radio-resistance and progression.

Pulmonary sarcomatoid carcinomas contain a sarcoma-like element (i.e. spindle or giant cells) associated with an epithelial component (i.e. adenocarcinoma, squamous cell carcinoma or poorly differentiated large-cell carcinoma) [1]. However, they are rare and their histology is poorly defined, so certain hypotheses regarding the development of sarcomatoid carcinoma, such as the collision of two independent cancers, or the transformation from carcinomatous to sarcomatous components and vice-versa, have been proposed [4–7]. The low frequency and histological heterogeneity of these tumors could account for the lack of studies on their carcinogenic mechanisms.

In this study, the epithelial and sarcomatous components in pulmonary sarcomatoid carcinoma were accurately separated by laser capture microdissection, and the mutation profiles were compared to determine whether the clonal origin of the components was identical. In addition, by utilizing *in silico* analysis, the evolutionary development of sarcomatoid cancer was estimated in order to identify the driver mutation suitable for treatment. Because genomic profiling is becoming increasingly crucial in the management of non-small cell lung cancer, new therapeutic strategies can be proposed based on our results related to the carcinogenesis of sarcomatoid cancer. Particularly, the clinical applicability of immune checkpoint blockade therapy, which has recently attracted attention as a treatment for non-small cell lung cancer was examined based on genomic and phenotypic characteristics.

## RESULTS

### Patient characteristics

Four patients who underwent surgery for sarcomatoid cancer were enrolled in this study. Three of these patients underwent lobectomy with or without chest wall resection as curative surgery and one patient underwent incisional biopsy because the tumor was unresectable (Table 1). The patient age ranged from 60 to 83 years (mean ± standard deviation [SD], 77.0 ± 7.0 years). There were three male and one female patients. The Brinkmann index (mean ± SD) was 1900 ± 818, and all four patients were smokers (Table 1).

**Table 1:** Patient characteristics

	Age	Sex	Smoking	B.I.	size (mm)	pTNM	pStage	Histology	Surgery	Outcome
Case 1	83	M	current	2520	110	pT4N0M0	IIIA	AdC+SaC	Lobectomy+Chest wall resection	3m, death
Case 2	78	F	current	780	47	pT3N0M0	IIB	AdC+SaC	Lobectomy+Chest wall resection	18m, no recurrence
Case 3	67	M	former	1800	55	pT4N2M0	IIIB	AdC+SaC	Biopsy	12m, stable disease
Case 4	80	M	former	2500	25	pT1cN0M0	IA3	SCC+SaC	Lobectomy	2m, no recurrence

B.I., Brinkman index; AdC, adenocarcinoma component; SaC, sarcomatous component; SCC, squamous cell carcinoma component; M, male; F, female; pStage, pathological stage; m, months.

**Figure 1:**
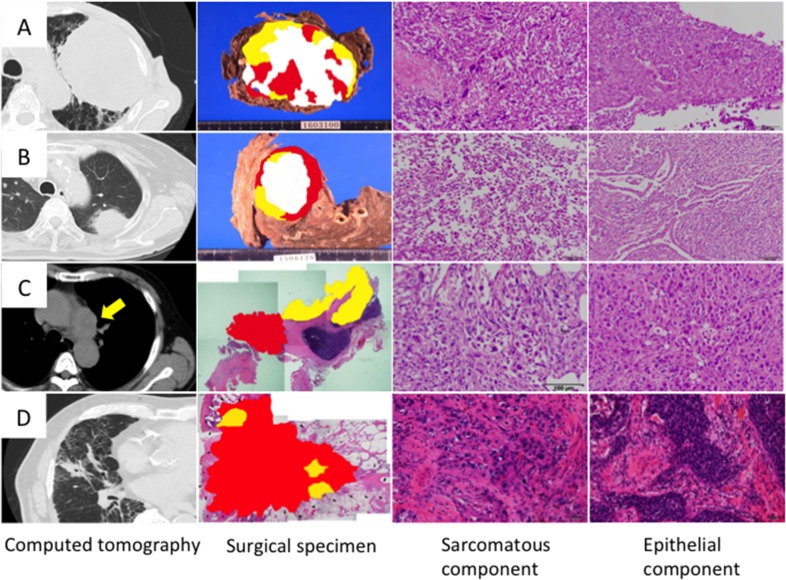
Histology of sarcomatoid cancer Cases 1, 2, 3 and 4 correspond to Figures **(A, B, C and D)**, respectively. Topographic presentation of the cut surface of the surgical specimens shows three different components; The carcinomatous and sarcomatous components are shown in yellow and red, respectively, and necrotized tissues are shown in white. Cancer cells were selected from carcinomatous and sarcomatous portions separately by laser capture microdissection.

Computed tomography showed that the maximum tumor diameter (mean ± SD) was 59.3 ± 36.1 mm (Table 1); most of the tumors were very large and had invaded the chest wall or mediastinum (Figure 1). On hematoxylin and eosin sections, the sarcomatous component was randomly distributed with no apparent pattern (Figure 1). Histologically, the epithelial component was adenocarcinoma in three cases and squamous cell carcinoma in one case, whereas the sarcomatous component was a mixture of spindle-shaped cells and giant cells in all cases (Figure 1). The postoperative pathological stages were IA3, IIB, IIIA and IIIB (Table 1).

### Determination of the sarcomatoid carcinoma mutation profiles by targeted deep sequencing

**Figure 2:**
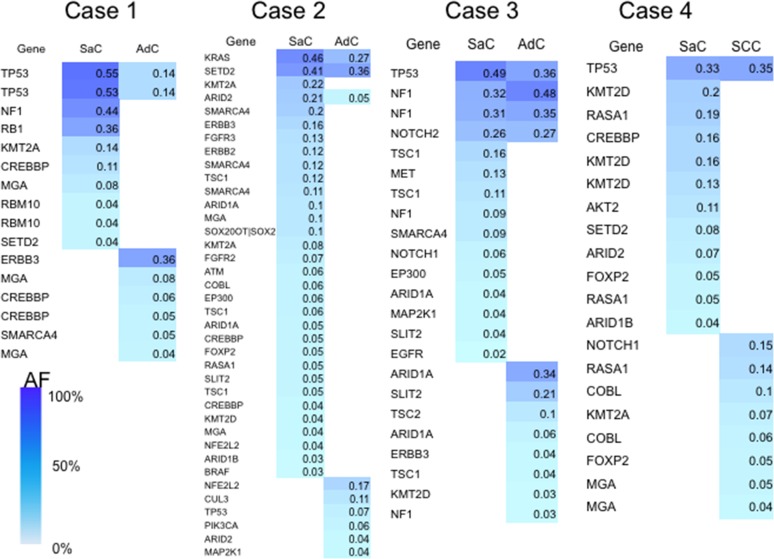
Heat map of the gene mutations This map shows the gene mutations of each component of the sarcomatoid cancers. The column next to the heat map shows the mutated genes. SaC, sarcomatous component; AdC, adenocarcinoma component; SCC, squamous cell carcinoma; AF, allele fraction.

Somatic mutations whose allele fractions were greater than 1% were regarded as significant and utilized for the analyses. As shown by the heat map, the carcinomatous and sarcomatous components had 1–4 common mutations (Figure 2). In contrast, the sarcomatous component had many mutations that were different from those of the carcinomatous component, and the number of mutations identified was greater in the sarcomatous component (Figure 2).

**Figure 3:**
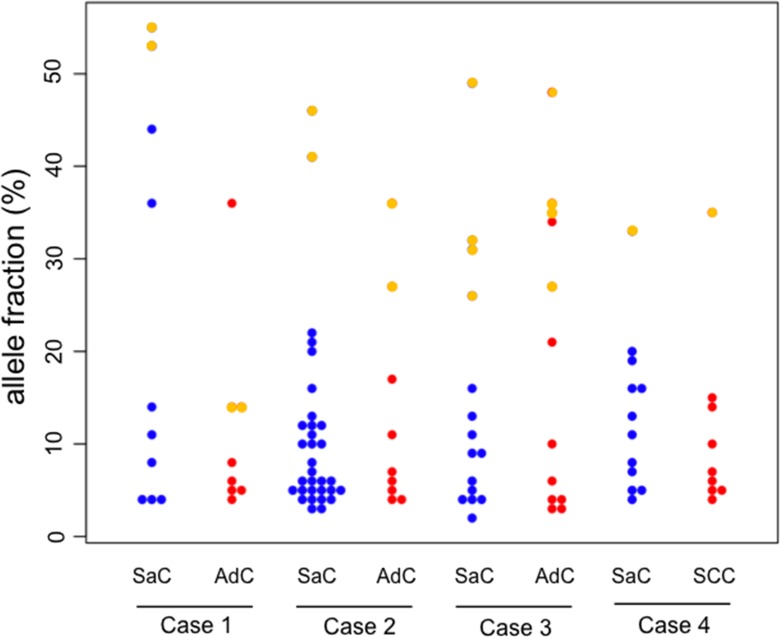
Beeswarm plot of the allele fraction of the gene mutations In the beeswarm plot, each colored dot represents a somatic mutation. Blue and red dots denote mutations which are exclusively present in the sarcomatous and adenocarcinoma component, respectively. Yellow points represent co-mutations which are shared by both histological components. SaC, sarcomatous component; AdC, adenocarcinoma component; SCC, squamous cell carcinoma.

A beeswarm plot for the allele fraction of the mutations in each histological component is presented in Figure 3 for visualization purposes. Several co-mutations clearly exist at relatively higher allele fraction in each component, while many mutations with low allele fraction were exclusively present in each component.

### PyClone analysis of sarcomatoid cancer based on targeted deep sequencing

**Figure 4:**
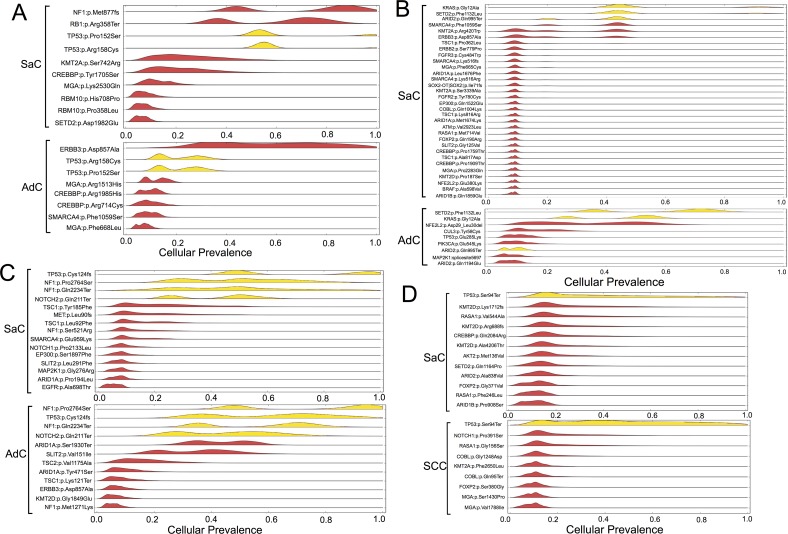
Statistical inference of the clonal population structure Case 1, 2, 3 and 4 corresponds to Figure **(A, B, C and D)**, respectively. The estimated cellular frequencies of the samples are shown as the distribution of posterior probabilities from the PyClone model. The red part represents the distribution of mutations harbored exclusively by each histological component. The yellow part represents the distribution of co-mutations shared by both components. SaC, sarcomatous component; AdC, adenocarcinoma component; SCC, squamous cell carcinoma.

PyClone is a Bayesian clustering method for grouping sets of deeply sequenced somatic mutations into putative clonal clusters while estimating their cellular prevalence and accounting for the allelic imbalances introduced by segmental copy number changes and normal cell contamination. PyClone analysis was performed based on the information obtained on genetic mutations. The analysis showed that the cellular prevalence of the common mutations between the carcinomatous and sarcomatous components was relatively high in all cases (Figure 4). It was inferred that the relatively major cell population in each component harbored several co-mutations. In contrast, in an additional experiment, samples were obtained from two different sites in the same tumor in two cases of adenocarcinoma as well as two cases of squamous cell carcinoma, and PyClone analysis was performed (Supplementary Figure 1). Each cancer sample harbored relatively few mutations, ranging from 2–7 (mean 3.9 ± 1.8), and mutations common to both sites were predominant (Supplementary Figure 1). In addition, in each of these tumors, homologous mutations were highly prevalent in two sites of the same tumor (Supplementary Figure 1). Thus, in these cancers displaying conventional histology, tumor cells had a relatively uniform mutation profile compared to the one observed in sarcomatoid cancers.

### Phylogenetic analysis of sarcomatoid cancer based on targeted deep sequencing

**Figure 5:**
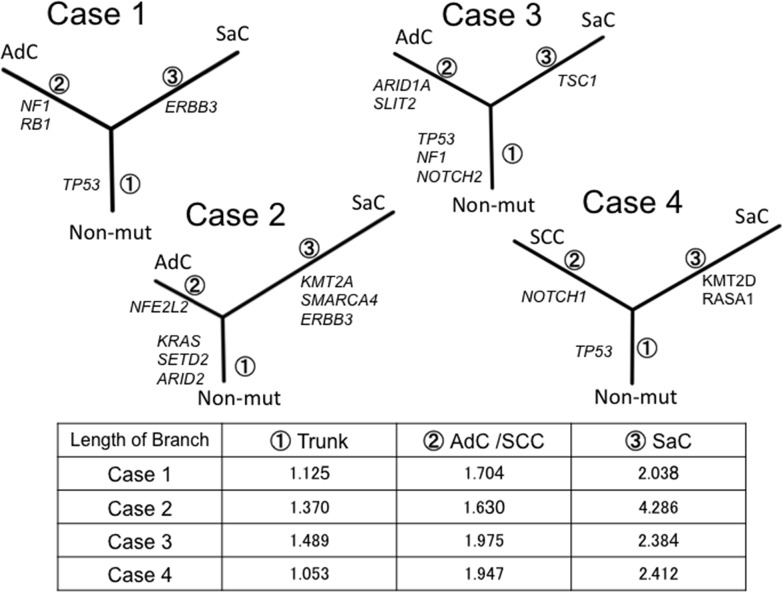
Phylogenetic analysis based on the targeted sequencing The phylogenetic tree was constructed using the bootstrap method. Potential mutations were acquired by the indicated genes in the branch. Branch length indicates the evolutionary distance and is correlated with the number of nucleotide substitutions. SaC, sarcomatoous component; AdC, adenocarcinoma component; SCC, squamous cell carcinoma; Non-mut, non-mutated cells.

For further inference of the phylogenies and estimation of the evolutionary distances, the neighbor-joining method was implemented to cluster the nonsilent mutations, and a phylogenetic tree was constructed (Figure 5). In all cases, the phylogenetic trees had similar topologies. Specifically, the trees had short trunks representing 2–4 common mutations, suggesting evolution from a common origin. Moreover, the branch representing the lineage to the sarcomatous component was longer than the one to the epithelial component. This finding reflects the fact that many mutations different from those in the epithelial component accumulated in the sarcomatous component.

### Phylogenetic analysis of sarcomatoid cancer based on whole exome sequencing

**Figure 6:**
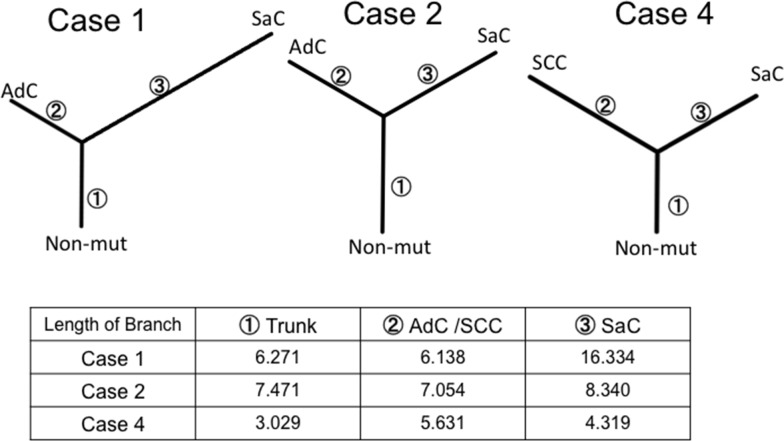
Phylogenetic analysis based on whole exome sequencing The phylogenetic tree was constructed using the bootstrap method. Branch length indicates the evolutionary distance and is proportional to the number of nonsynonymous mutations. SaC, sarcomatous component; AdC, adenocarcinoma component; SCC, squamous cell carcinoma; Non-mut, non-mutated cells.

In order to better characterize the biology of sarcomatoid carcinoma, we performed whole exome sequencing in cases 1, 2 and 4. This analysis was not performed in case 3 because of the insufficient amount of input DNA. Phylogenetic trees similar to those based on targeted deep sequencing were created for cases 1, 2 and 4. The sarcomatous component accumulated a large number of unique mutations, indicating that this component was substantially different from the epithelial component in terms of genetic status (Figure 6).

### Genomic and immunohistochemical evaluation of microsatellite instability

**Figure 7:**
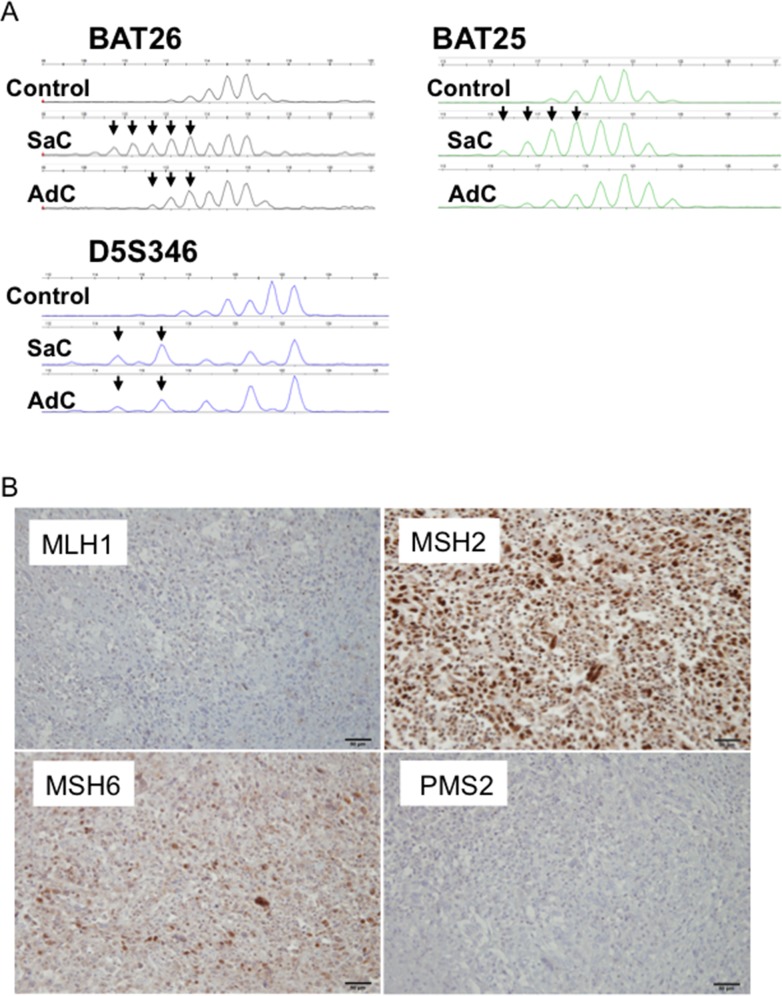
MSI profiles in Case 1 **(A)** Size variation in the number of microsatellite repeats for BAT25, BAT26 and D5S346 in Case 1. A peripheral blood sample was used as a control. SaC, sarcomatous component; AdC, adenocarcinoma component. **(B)** Mismatch repair protein expression in Case 1. MLH1 and PMS2 were not expressed.

Whole exome sequencing revealed somatic mutations in the MutL homolog 1 (*MLH1)* gene in Case 1 (Table 2, Supplementary Table 1). This *MLH1^K311fs^* mutation was a frame-shift mutation, leading to loss of mismatch repair function. Both the carcinomatous and sarcomatous components harbored this somatic mutation (Supplementary Table 1). Microsatellite instability (MSI) analysis revealed that the cancers in Cases 2-4 exhibited microsatellite stability, whereas two and three markers were positive for adenocarcinoma and sarcomatous components in Case 1, respectively (Figure 7A, Table 3). Thus, both the adenocarcinomatous and sarcomatous components in Case 1 displayed a high level of MSI. Immunohistochemically, MLH1, MutS protein homolog (MSH) 2, MSH6 and Postmeiotic segregation increased 2 (PMS2) were all expressed in the nuclei of tumor cells in Cases 2 and 3, whereas MLH1 and PMS2 were not expressed in Case 1, suggesting the mismatch repair function had diminished in Case 1 (Figure 7B, Table 3). There was no significant difference in the staining intensity between the sarcomatous and adenocarcinoma components (data not shown). Altogether, whole exome sequencing, MSI analysis and immunohistochemical staining consistently supported microsatellite instability in Case 1 (Table 3).

**Table 2:** Mutation burden and MMR genes mutation detected by next generation sequencing

	Mutation burden	Mutation burden	Mutation of MMR genes
	<panel>	<wes>	
	(EpC/SaC/total)	(EpC/SaC/total)	
Case 1	8/10/16	154/511/665	*MLH-1*
Case 2	9/32/39	221/395/616	None
Case 3	12/15/23	-	-
Case 4	8/12/19	85/72/117	None

WES, whole exome sequence; EpC, epithelial component; SaC, sarcomatous component; MMR, mismatch repair; -, not examined.

**Table 3:** Evaluation of microsatellite instability status

Sample	BAT25	BAT26	D17S250	D2S123	D5S346	Number of markers	MSI status	MMR genes mutations	MLH1 IHC	MSH2 IHC	MSH6 IHC	PMS2 IHC
Case 1 AdC	-	+	-	-	+	2	MSI-High	*MLH1* Somatic Mutation	Negative	Positive	Positive	Negative
Case 1 SaC	+	+	-	-	+	3	MSI-High	*MLH1* Somatic Mutation	Negative	Positive	Positive	Negative
Case 2 AdC	-	-	-	-	-	-	MSS	None	Positive	Positive	Positive	Positive
Case 2 SaC	-	-	-	-	-	-	MSS	None	Positive	Positive	Positive	Positive
Case 3 AdC	-	-	-	-	-	-	MSS	ND	Positive	Positive	Positive	Positive
Case 3 SaC	-	-	-	-	-	-	MSS	ND	Positive	Positive	Positive	Positive
Case 4 SCC	-	-	-	-	-	-	MSS	None	Positive	Positive	Positive	Positive
Case 4 SaC	-	-	-	-	-	-	MSS	None	Positive	Positive	Positive	Positive

Pathogenetic findings are highlighted in red.

AdC, adenocarcinoma component; SaC, sarcomatous component; SCC, squamous cell carcinoma; MSI, microsatellite instability; MSS, microsatellite stable; MMR, mismatch repair; IHC, immunohistochemistry; ND, not determined.

### Immunohistochemical staining of programmed death ligand-1 (PD-L1)

**Figure 8:**
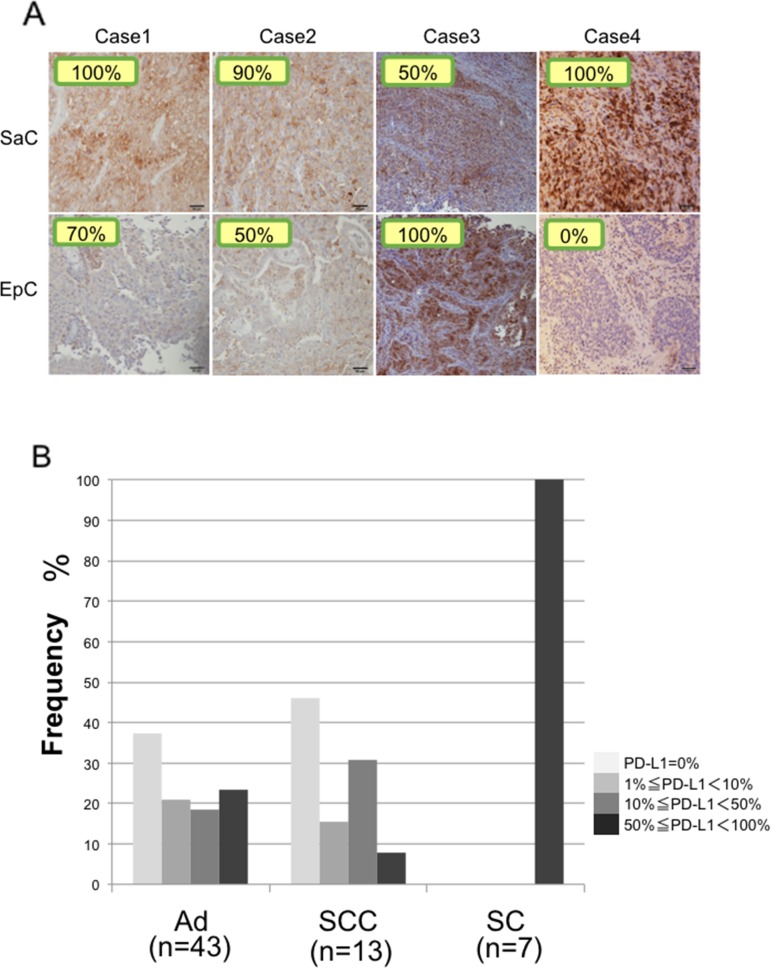
Immunohistochemical staining of PD-L1 **(A)** PD-L1 expression in the sarcomatous and epithelial components in all cases. The staining level is quantitatively shown in the image. SaC, sarcomatous component; EpC, epithelial component. **(B)** Proportions of patients with PD-L1 expression in adenocarcinoma, squamous cell carcinoma and sarcomatoid carcinoma. Ad, adenocarcinoma; SCC, squamous cell carcinoma; SC, sarcomatoid cancer.

In all four patients, PD-L1 expression was observed on the membrane of tumor cells in both the epithelial and sarcomatous components (Figure 8A). The staining intensity of PD-L1 expression was 85.0 ± 23.8 % and 55.0 ± 42.0 % in the sarcomatous and epithelial components, respectively (Figure 8A). PD-L1 expression did not significantly differ between the two components (*p* = 0.26). There was no significant correlation between PD-L1 staining and mutation burden (*p* = 0.49).

## DISCUSSION

Pulmonary sarcomatoid carcinoma is a small subgroup of poorly differentiated tumors containing sarcoma-like elements; however, the carcinogenesis of these tumors remains unclear. Pulmonary sarcomatoid carcinomas may undergo a dedifferentiation process with activation of an epithelial-mesenchymal transition [4]. These phenotypic changes to sarcomatoid cancer are thought to be associated with the addition of unique mutation patterns [6]. In this study, we were able to analyze the mutations of the two components in pulmonary sarcomatoid carcinoma separately and comprehensively utilizing bioinformatics in combination with laser capture microdissection, next generation sequencing and *in silico* analysis. This enabled investigation of the carcinogenic process underlying pulmonary sarcomatoid carcinoma from the perspective of genome evolution. The results supported the hypothesis that the components have a common origin [5–7]. In addition, comparison of the mutation profile in each component showed that the genomic distance between carcinomatous and sarcomatous components increased following the accumulation of additional mutations. Furthermore, the sarcomatous component showed a greater accumulation of mutations and a larger genetic distance to the common-origin than the epithelial component. These genetic characteristics may explain why sarcomatoid carcinoma is high-grade and resistant to conventional chemotherapy.

The neighbor-joining method was applied to analyze complex models of nucleotide substitutions for the purpose of estimating evolutionary distance and to infer the phylogeny of the sarcomatoid cancer [8]. Neighbor-joining is one of the most popular distance-based reconstruction techniques and is an agglomerative clustering approach in which at each stage two neighboring elements are joined to one cluster; this new cluster then replaces them in the set of elements, and the clustering continues recursively on this reduced set [8]. Thus, we established sarcomatoid cancer phylogenetic trees that exhibited a similar structure, regardless of whether panel sequencing or whole exome sequencing was performed. In addition, *in silico* analysis led to the following reasoning regarding treatment. First, trunk mutations are thought to be present in many cell populations in both components and function as putative driver mutations; therefore, molecular targeted therapy against trunk mutations would be expected to be effective. Second, considering the many mutations in the branches of the phylogenetic trees, treatment with anti-PD-1/PD-L1 therapies would also be expected to be effective [9].

An analysis was performed on patients who had undergone targeted deep sequencing in our department, including those with adenocarcinoma, squamous cell carcinoma and sarcomatoid cancer; the numbers of mutations with an allele frequency of greater than 1% were compared among these three groups. The mutation burden in sarcomatoid carcinoma was significantly higher than that in adenocarcinoma and squamous cell carcinoma (Supplementary Figure 2). In several cohort studies, higher nonsynonymous mutation burden in tumors was associated with improved objective response, durable clinical benefit and progression-free survival in anti-PD-1/PD-L1 therapies [10]. Tumors with a high number of mutation-associated neoantigens may be more likely to stimulate the immune system to react against the tumor mutation burden. In some cases, patients with renal cell carcinoma with sarcomatoid features were found to respond to anti-PD-1 therapy [11, 12].

Mismatch repair (MMR) is an important type of DNA repair and plays a pivotal role in maintaining genome stability [13]. The MMR genes (*MLH1*, *MSH2*, *MSH6* and *PMS2*), which are key components of the MMR system, recognize and excise single-base mismatches and insertion/deletion loops that occur during DNA replication or DNA damage [14]. MMR dysfunction often leads to genomic instability, including microsatellite instability and the accumulation of gene mutations that are thought to be associated with various malignant tumors [15, 16].

MMR-deficient colorectal cancers have 10–100 times as many somatic mutations as MMR-proficient colorectal cancers [17, 18]. MMR-deficient colorectal cancers exhibit different clinical behavior from those of MMR-proficient cancers, and MMR status predicts the clinical benefit of an anti-PD-1 immune checkpoint inhibitor [10]. MMR deficiency occurs in several types of cancers, including those of the colorectum, uterus, stomach, biliary tract, pancreas, ovary, prostate and small intestine [18–26]. However, it is not clear whether MMR affects the gene mutations in non-small cell lung cancer. Data on the prevalence of microsatellite instability among non-small cell lung cancers are conflicting, with estimates ranging between 0% and 0.8% [27, 28], and the clinical relevance of microsatellite instability is largely unknown.

Hereditary nonpolyposis colorectal cancer (also known as Lynch syndrome) results from an inherited germline defect in one of four MMR genes (*MLH1*, *MSH2*, *MSH6* and *PMS2*) followed by a second inactivating somatic change in the remaining wild-type allele. However, our case (Case 1) was thought to be a sporadic MMR-deficient tumor in which both alleles of the *MLH1* gene were inactivated by somatic mutation.

Pulmonary sarcomatoid cancers are associated with poor prognosis and high levels of chemoresistance, highlighting the necessity for the identification of potential new therapies. New targets must therefore be explored in order to improve the management of these tumors. Therapies targeting immune molecule checkpoints could be particularly interesting in this regard. PD-1 is a co-inhibitory inducible receptor present on T cells and macrophages [29]. Tumor cells with increased PD-L1 expression are believed to escape immunity through activation of the PD-1/PD-L1 pathway and suppression of the effector-immune responses [29]. Recent strategies targeting the PD-1/PD-L1 axis have shown promising results in patients with several tumors types, including lung carcinomas [30–32]. Recent randomized controlled trials have suggested that PD-L1 protein expression may have a predictive response to such therapies [33]. Comparisons of PD-L1 staining were performed in patients who underwent PD-L1 staining in our department, including patients with adenocarcinoma, squamous cell carcinoma and sarcomatoid cancer. PD-L1 staining was also performed in three surgical cases of sarcomatoid cancer treated in 2007–2013, so the total number of cases of sarcomatoid cancer examined by PD-L1 staining was seven. The results showed that PD-L1 was expressed in 50% or more of cancer cells in all patients with sarcomatoid cancer, and the proportion of patients with strong staining was significantly greater in sarcomatoid cancer than in adenocarcinoma and squamous cell carcinoma (Figure 8B). Our findings support the potential value of anti-PD-1/PD-L1 targeted therapies.

This study has a few limitations. The sample sizes were extremely small due to the rare occurrence of sarcomatoid cancer. Therefore, further studies are warranted to confirm our results. Nevertheless, our findings clearly supported the possibility of using novel immunotherapy approaches, such as PD-1/PD-L1 blockers, in this otherwise difficult-to-treat disease. We anticipate that the use of PD-L1 or PD-1 immunotherapy in this histological subtype will be validated by prospective and comparative studies in the future.

## MATERIALS AND METHODS

### Patients and sample preparation

Four patients who had undergone surgery in our department for pulmonary sarcomatoid cancers between July 2014 and March 2016 were enrolled in this study. All participants provided written informed consent prior to participation in the genetic research. The research was conducted in accordance with the Declaration of Helsinki, and the study was approved by the Institutional Review Board Committee of Yamanashi Central Hospital (Yamanashi, Japan).

Serial sections of formalin-fixed, paraffin-embedded (FFPE) tissues were stained with hematoxylin-eosin. To examine whether different histological components resulted from genetic divergence, tumor cells of the epithelial and sarcomatous areas were collected from FFPE tissues using an ArcturusXT laser-capture microdissection system (Thermo Fisher Scientific Waltham, MA).

DNA was extracted using a QIAamp DNA FFPE Tissue Kit (Qiagen, Hilden, Germany). FFPE DNA quality was checked using primers for the ribonuclease P (RNase P) locus [34]. A peripheral blood sample was collected from each patient just prior to surgery. The buffy coat was isolated following centrifugation, and DNA was extracted from the buffy coat using a QIAamp DNA Blood Mini Kit with a QIAcube system (Qiagen).

### Targeted deep sequencing and data analysis

A panel targeting the exons of 53 lung cancer-associated genes (Supplementary Table 2) was established to perform targeted sequencing. We searched the literature and selected these genes based on the following criteria: (a) genes often involved in lung cancer from The Cancer Genome Atlas [35, 36] and other projects [37–41] or (b) genes frequently mutated in lung cancer from the COSMIC database (http://cancer.sanger.ac.uk/cancergenome/projects/cosmic). Primer design for the targeted sequencing was performed using Ion AmpliSeq designer software (Thermo Fisher Scientific), as previously reported [42–44]. Sequencing libraries were prepared using an Ion AmpliSeq Library kit (Thermo Fisher Scientific) according to the manufacturer's instructions. After barcode ligation using an Ion Xpress Barcode Adapters kit (Thermo Fisher Scientific), library samples were purified using Agencourt AMPure XP reagent (Beckman Coulter, Brea, CA) and subsequently quantified using an Ion Library Quantitation Kit (Thermo Fisher Scientific). Emulsion PCR and chip loading were performed on the Ion Chef with the Ion PI Hi-Q Chef kit. Sequencing was performed using an Ion PI Hi-Q Sequencing Kit on the Ion Proton Sequencer (Thermo Fisher Scientific).

The sequence data were processed using standard Ion Torrent Suite Software running on the Torrent Server. Raw signal data were analyzed using Torrent Suite version 5.0.4. The pipeline included signaling processing, base calling, quality score assignment, read alignment to the human genome 19 reference (hg19), quality control of mapping and coverage analysis. Following data analysis, annotation of single nucleotide variants, insertions and deletions was performed using an Ion Reporter Server System (Thermo Fisher Scientific), and lymphocytes from peripheral blood DNA were used as a control to detect variants (Tumor-Normal pair analysis) [45]. Sequence data were visually confirmed with the Integrative Genomics Viewer. Beeswarm plots were produced using R package graphics and Beeswarm.

### Whole exome sequencing

Whole exome sequencing and multiplex PCR were performed using buffy coat DNA and tumor DNA with an Ion AmpliSeq Exome RDY Kit (Thermo Fisher Scientific). The pooled PCR amplicons were treated with FuPa reagent to partially digest the primer sequences and phosphorylate the amplicons. The amplicons were ligated to adapters with the diluted barcodes of the Ion Xpress Barcode Adapters kit (Thermo Fisher Scientific).

Adaptor-ligated amplicon libraries were purified using Agencourt AMPure XP reagents (Beckman Coulter). Each library was diluted and the same amount of each library was pooled for a single sequence reaction. Emulsion PCR and chip loading was performed on the Ion Chef with the Ion PI Hi-Q Chef kit. Sequencing was performed using an Ion PI Hi-Q Sequencing Kit on the Ion Proton Sequencer (Thermo Fisher Scientific).

### *In silico* analysis

For further inference of the phylogenies and estimation of the evolutionary distances, the neighbor-joining method was implemented to cluster the nonsilent mutations, and a phylogenetic tree was constructed [46]. The ‘ape’ and ‘phangon R’ (3.2.3 on linux) packages were utilized for these analyses.

To estimate the fraction of cancer cells harboring a mutation, PyClone analysis was performed [47]. Mutations whose allele fractions were greater than 1% were regarded as significant and were utilized for *in silico* analyses.

### Microsatellite instability analysis

For MSI analysis, five microsatellite markers (BAT25, BAT26, D5S346, D2S123 and D17S250) were used to classify the tumor as MSI-high (the presence of at least two markers showing novel alleles compared with normal control), MSI-low (defined as one marker with a novel allele), or microsatellite stable (MSS, no marker with novel alleles) [48]. Electrophoretic mobility in these microsatellites from tumors and matched peripheral blood samples was compared. Fluorescently labeled PCR products were separated by capillary electrophoresis using a 3500 Genetic Analyzer (Applied Biosystems) and the product size was analyzed with GeneMapper Software 5 (Applied Biosystems). The primer sequences are provided in Supplementary Table 3 [49].

### Immunohistochemistry for MMR disturbance and immune-checkpoint PD-L1

FFPE tissue sections were cut in 5-μm-thick slices, deparaffinized and rehydrated. All slides were sectioned within 2 months of staining. Immunohistochemistry analysis was performed on tumor samples using commercially available detection kits, automated staining techniques (Ventana Benchmark ULTRA system; Roche, Tucson, AZ), and antibodies against MLH1 (M1; Ventana), MSH2 (G219-1129; Ventana), MSH6 (44; Ventana), PMS2 (EPR3947; Ventana) and PD-L1 [8–28] (ab205921;Abcam, at 1/250 dilution). Microsatellite instability was assessed using immunostaining patterns for the four genes involved in MMR (*MLH1*, *MSH2*, *MSH6* and *PMS2*). In the normal state, the four proteins are supposed to be diffusely expressed; however, in the presence of abnormalities, the expression of these proteins is abolished. Immunohistochemistry is reported to provide a rapid, cost-effective, sensitive (92.3%) and extremely specific (100%) method for the screening of DNA mismatch repair defects [50]. Samples were dichotomized as having positive or negative staining for each MMR protein. For PD-L1, the expression was evaluated on a quantitative scale, from 0% to 100%, by two pathologists.

### Statistical analysis

Continuous variables are presented as means ± SDs and compared using the unpaired Student's *t*-test. One-way analysis of variance and Tukey-Krammer multiple comparisons tests were used to detect significant differences between groups. Chi-square tests were used to compare the categorical data between groups. Correlations between samples were calculated using Pearson's correlation coefficient. All statistical analyses were performed using the JMP function in the SAS software package (SAS Institute, Inc., Cary, NC, USA). *P* values of less than 0.05 in two-tailed analyses were considered to denote statistical significance.

## SUPPLEMENTARY MATERIALS FIGURES AND TABLES



## Abbreviations

MMR: mismatch repair
MSI: microsatellite instability
MLH1: MutL homolog 1
MSH2: MutS protein homolog 2
MSH6: MutS protein homolog 6
PMS2: postmeiotic segregation increased 2
FFPE: formalin-fixed paraffin-embedded
