# Supplementary material for: New therapeutic targets for pulmonary sarcomatoid carcinomas based on their genomic and phylogenetic profiles
Source: Oncotarget. 2018 Jan 31;9(12):10635–49. doi: 10.18632/oncotarget.24365 (PMC5828205; doi:10.18632/oncotarget.24365)
Supplement: Supplementary file 1 [file oncotarget-09-10635-s001.pdf]

New therapeutic targets for pulmonary sarcomatoid carcinomas based on their genomic and phylogenetic profiles

SUPPLEMENTARY MATERIALS

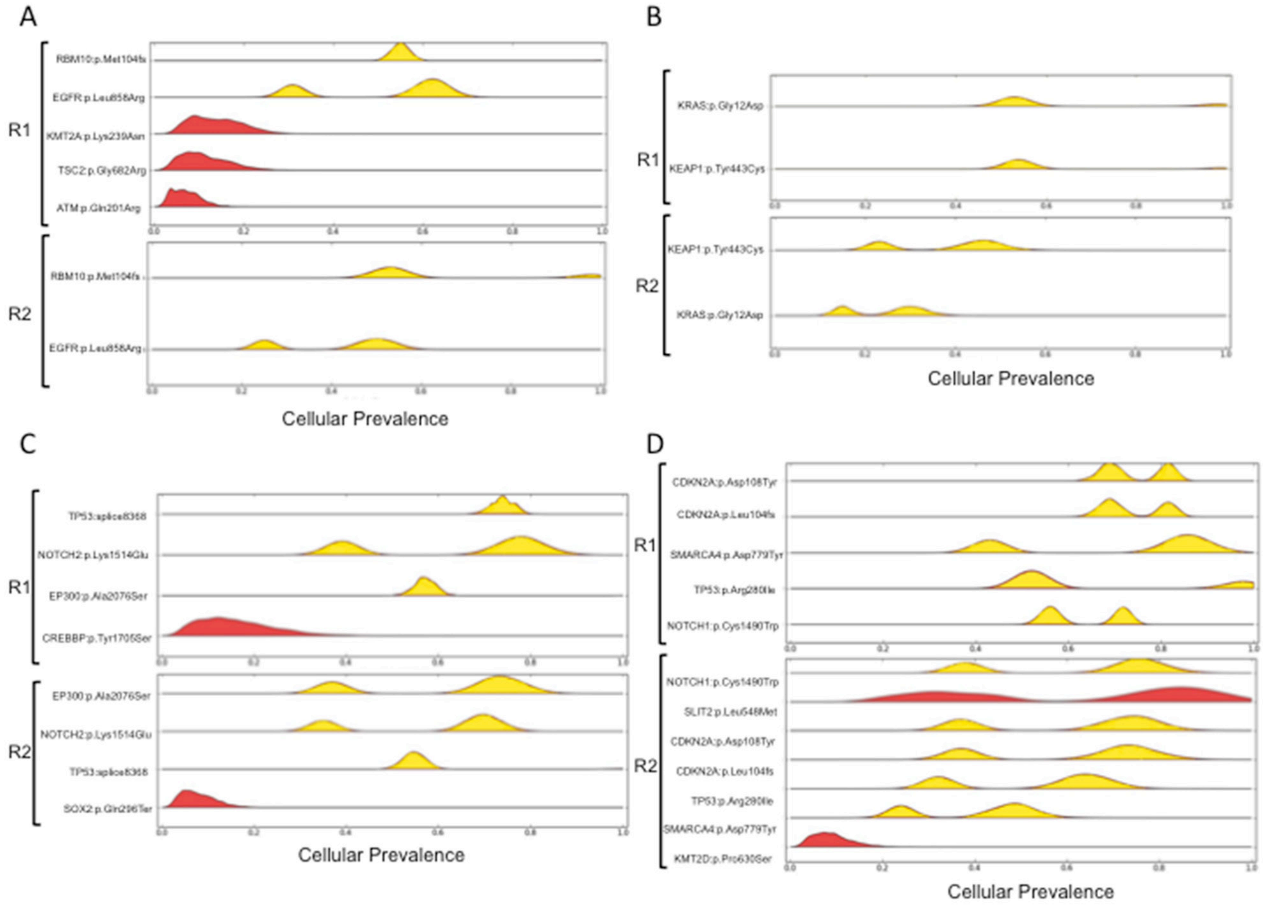

Supplementary Figure 1: PyClone analysis of adenocarcinoma and squamous cell carcinoma based on targeted deep sequencing. To examine lung cancer exhibiting conventional histology, samples were obtained from two different sites (Regions 1 and 2) of the same tumor in two cases of adenocarcinoma **(A** and **B)** and two cases of squamous cell carcinoma **(C** and **D)**; these samples were subjected to the targeted deep sequencing and PyClone analysis. The estimated cellular frequencies of the samples are shown as the distribution of posterior probabilities from the PyClone model. The red part represents the distribution of mutations harbored exclusively by each histological component. The yellow part represents the distribution of co-mutations shared by both components. R, region.

Supplementary Table 1: Somatic mutation of *MLH1* gene in Case 1

Sample	Locus	Type	Ref.	Var.	Coverage	AF	Gene	Protein	Coding
SaC	chr3:37061846	Indel	AAAGCATGAAGT TCACTTCCTGC	A	101	54%	*MLH1*	p.Lys311frame-shift	c.931_952delAAGCATG AAGTTCACTTCCTGC
AdC	chr3:37061846	Indel	AAAGCATGAAGT TCACTTCCTGC	A	65	25%	*MLH1*	p.Lys311frame-shift	c.931_952delAAGCATG AAGTTCACTTCCTGC

SaC, sarcomatous component; AdC, adenocarcinoma component; Ref., reference; Var., variance; AF, allele fraction.

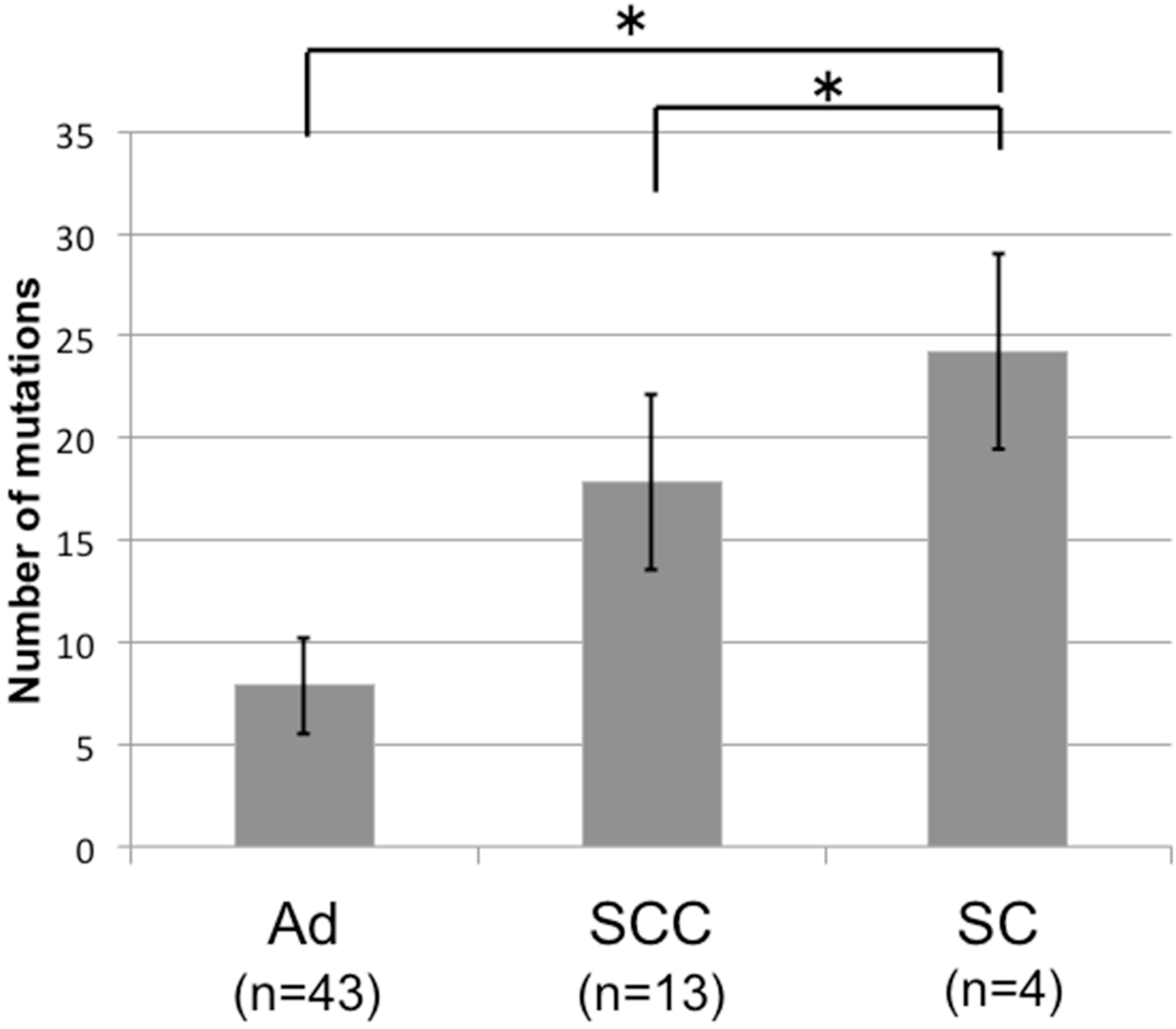

Supplementary Figure 2: Mutation burden in sarcomatoid cancer. Sarcomatoid carcinomas harbor larger number of mutations, compared to adeno- or squamous cell carcinomas. Ad, adenocarcinoma; SCC, squamous cell carcinoma; SC, sarcomatoid cancer. *, $p < 0.05$.

Supplementary Table 2: The genes targeted in the cancer panel

No	Gene symbol	Chromosome	Number of amplicons	Total bases	Covered bases	Overall coverage
1	AKT1	chr14	26	1573	1497	95%
2	AKT2	chr19	27	1576	1543	98%
3	AKT3	chr1	30	1624	1624	100%
4	ARID1A	chr1	76	7058	6023	85%
5	ARID1B	chr6	75	6950	5965	86%
6	ARID2	chr12	71	5718	5643	99%
7	ASCL4	chr12	5	532	382	72%
8	ATM	chr11	147	9791	9439	96%
9	BRAF	chr7	37	2481	2224	90%
10	CDKN2A	chr9	9	962	612	64%
11	COBL	chr7	48	4151	3977	96%
12	CREBBP	chr16	96	7639	7071	93%
13	CTNNB1	chr3	32	2486	2486	100%
14	CUL3	chr2	42	2561	2495	97%
15	EGFR	chr7	60	4189	4135	99%
16	EP300	chr22	90	7555	7182	95%
17	EPHA7	chr6	44	3175	3154	99%
18	ERBB2	chr17	57	4080	3808	93%
19	ERBB3	chr12	59	4440	4374	99%
20	FGFR1	chr8	41	2825	2816	100%
21	FGFR2	chr10	43	2910	2842	98%
22	FGFR3	chr4	34	2752	2215	81%
23	FOXP2	chr7	36	2487	2469	99%
24	HRAS	chr11	11	683	683	100%
25	KEAP1	chr19	24	1925	1845	96%
26	KMT2D	chr12	192	17154	15854	92%
27	KRAS	chr12	10	737	681	92%
28	MAP2K1	chr15	18	1292	1239	96%
29	MET	chr7	59	4427	4396	99%
30	MGA	chr15	110	9428	9345	99%
31	MLL	chr11	144	12279	11875	97%
32	NF1	chr17	136	9161	9023	99%
33	NFE2L2	chr2	23	1868	1826	98%
34	NOTCH1	chr9	99	8008	7078	88%

(Continued)

No	Gene symbol	Chromosome	Number of amplicons	Total bases	Covered bases	Overall coverage
35	NOTCH2	chr1	101	7809	7539	97%
36	NRAS	chr1	9	610	610	100%
37	PIK3CA	chr3	50	3407	3282	96%
38	PTEN	chr10	18	1302	1223	94%
39	RASA1	chr5	55	3412	3216	94%
40	RB1	chr13	55	3057	2902	95%
41	RBM10	chrX	48	3228	3079	95%
42	RIT1	chr1	13	771	771	100%
43	SETD2	chr3	91	7905	7663	97%
44	SLIT2	chr4	76	4972	4854	98%
45	SMAD4	chr18	24	1769	1715	97%
46	SMARCA4	chr19	74	5399	5055	94%
47	SOX2	chr3	9	964	883	92%
48	STK11	chr19	23	1392	1343	97%
49	TP53	chr17	22	1383	1351	98%
50	TP63	chr3	34	2360	2227	94%
51	TSC1	chr9	49	3705	3603	97%
52	TSC2	chr16	92	5834	5677	97%
53	U2AF1	chr21	15	880	870	99%

Supplementary Table 3: Primer sequencings for MSI analysis

Primer name	Sequencing	Application
D2S123 For	5′-NED-AAACAGGATGCCTGCCTTTA-3′	MSI
D2S123 Rev	5′-GGACTTTCCACCTATGGGAC-3′	MSI
D17S250 For	5′-FAM-GGAAGAATCAAATAGACAAT-3′	MSI
D17S250 Rev	5′-GCTGGCCATATATATATATTTAAACC-3′	MSI
D5S346 For	5′-FAM-ACTCACTCTAGTGATAAATCGGG-3′	MSI
D5S346 Rev	5′-AGCAGATAAGACAGTATTACTAGTT-3′	MSI
BAT25 For	5′-VIC-TCGCCTCCAAGAATGTAAGT-3′	MSI
BAT25 Rev	5′-TCTGCATTTTAACTATGGCTC-3′	MSI
BAT26 For	5′-NED-TGACTACTTTTGACTTCAGCC-3′	MSI
BAT26 Rev	5′-AACCATTCAACATTTTTAACCC-3′	MSI

MSI, microsatellite instability.
